# A retrospective study of lymphatic transverse rectus abdominis myocutaneous/deep inferior epigastric perforator flaps for breast cancer treatment-induced upper-limb lymphoedema

**DOI:** 10.1038/s41598-017-00164-1

**Published:** 2017-03-06

**Authors:** Zhuangqing Yang, Sheng Huang, Jiankui Wang, Yan Xi, Xiaojuan Yang, Qi Tang, Juan Du, Jianyun Nie, Tianning Zou, Shaoqing Zhou, Xueliang Tang, Dedian Chen

**Affiliations:** 1 The 2nd Department of Breast Surgery, Breast Cancer Center of the Third Affiliated Hospital of Kunming Medical University, Yunnan province, China; 2 Department of Head and Neck Surgery, the Third Affiliated Hospital of Kunming Medical University, Yunnan province, China

## Abstract

Breast cancer-related lymphoedema (BCRL) is a common and intractable complication. To evaluate the possible complications of using lymphatic transverse rectus abdominis myocutaneous/deep inferior epigastric perforator (TRAM/DIEP) flaps for breast reconstruction and BCRL treatment, 20 patients with moderate or severe BCRL were retrospectively enrolled between November 2012 and October 2014. 10 patients had undergone lymphatic TRAM/DIEP flap surgery were assigned to the surgery group. 10 patients unwilling to undergo reconstruction were assigned to the physiotherapy group treated with traditional physical therapy. Upper-limb movement and circumference were measured and patients’ subjective assessment was assessed using a questionnaire. In the surgery group, all flaps were successfully transferred. BCRL in 8 patients was improved by one level. The upper-limb circumference returned to normal in 1 case, and only 1 patient did not improve. In the physiotherapy group, a slight improvement was noted in 6 patients and unchanged in four cases. From the questionnaires, patients underwent lymphatic TRAM/DIEP flap surgery reported a significantly greater improvement in the affected limb (*p* < 0.05). In the physiotherapy group, the limb subjective did not improve as well as in the surgery group. Lymphatic TRAM/DIEP is a safe and effective option for patients who suffer from post-mastectomy lymphoedema.

## Introduction

Globally, breast cancer is the most commonly occurring cancer among women, comprising 23% of the ~1.7 million female cancers that are newly diagnosed each year^1^. Approximately 6.2 million women have been diagnosed with breast cancer in the last 5 years, making breast cancer the single most prevalent cancer around the globe^1^. Although treatment for breast cancer has improved, arm lymphoedema following axillary lymph node dissection (ALND) remains a common complication, occurring in 31–83% of patients who undergo this treatment^2–4^. Affected patients develop a chronic accumulation of interstitial fluid, resulting in fibro-adipose deposition and swelling. This swelling can lead to pain, decreased function, body image disturbance, and anxiety.

Breast cancer-related lymphoedema (BCRL) treatment remains challenging. Currently, surgical and non-surgical strategies are somewhat effective for BCRL, but with these treatments, the possibility of a cure remains uncertain. Using microsurgical techniques, we have used transverse rectus abdominis myocutaneous/deep inferior epigastric perforator (TRAM/DIEP) flaps combined with tissue surrounding the superficial circumflex iliac vessels, particularly including lymph nodes, to reconstruct the breast and treat BCRL. The purpose of this study was to evaluate the efficacy of lymphatic TRAM/DIEP flaps for treating upper-limb lymphoedema after breast cancer surgery.

## Results

**Figure 1:**
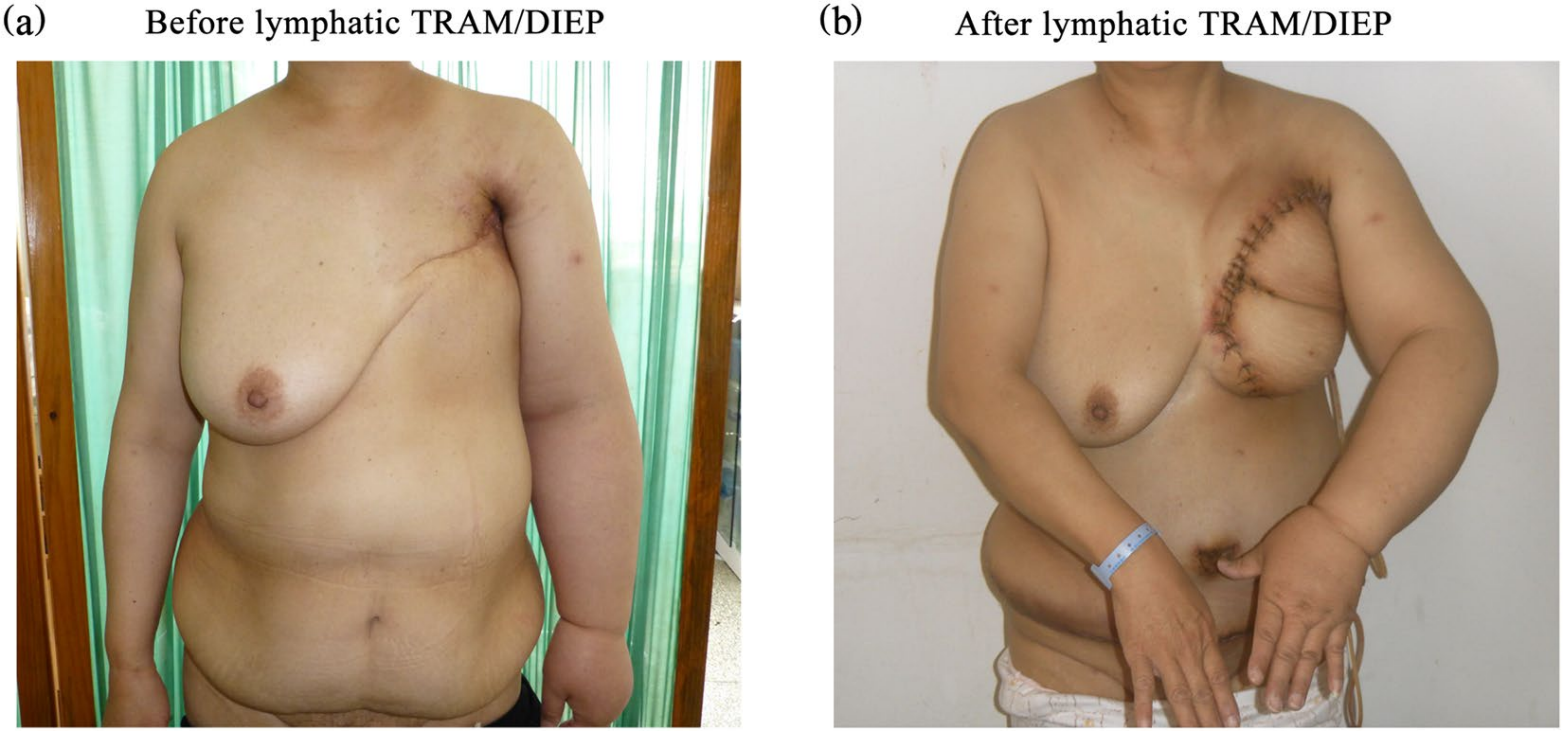
Preoperative evaluation and postoperative 2-week evaluation. (**a**) Preoperative status before lymphatic TRAM/DIEP. (**b**) Postoperative 2-week evaluation.

There were 4 patients with moderate oedema and 6 patients with severe oedema enrolled in the surgery group with a corresponding number of patients with the same degree of oedema enrolled in the physiotherapy group. All 20 patients accepted modified radical mastectomy, and 9 in each research group undergone radiotherapy. In the surgery group, all flaps worked well. None of them lost either a whole or part of flap. Fat necrosis occurred in the lower inner quadrant in 1 patient’s flap. For this patient, the size of the fat necrosis was 3 × 2.8 × 2.5 cm. 6 points circumferences of the affected upper limb measured in both two groups before and after BCRL treatment. Comparing to the baseline data before surgery (Table 1), after lymphatic TRAM/DIEP flap surgery, limb oedema significantly improved during follow-up (*p* < 0.05), especially in the first week (Fig. 1). In the surgery group, except for the circumference of the elbow through the olecranon, the circumferences of the other 5 points after the first week following the surgery were enlarger slightly. However, even in this condition, compared to the baseline, oedema in the affected limb showed good improvement. The mean reduction is described in Table 1. Oedema in the affected limb improved to the next-better level in 8 patients by the end of the first year, including in 5 patients with severe oedema and 3 with moderate oedema. And 1 patient with moderate oedema returned to normal limb. Patients in the physiotherapy group experienced none of therapeutic effect like lymphatic TRAM/DIEP flaps. 6 points circumferences of the affected upper limbs in the physiotherapy group are described in Table 2. The circumferences of the limbs in the physiotherapy group did not decrease to the extent that they did in the surgery group. Even after physiotherapy, the oedema of the affected limbs in the physiotherapy group worsened in the first year.

**Table 1:** The circumference of the affected upper limb after lymphatic TRAM/DIEP ($\overline{{\rm{x}}}$  ± s, cm).

The 6 points	Before surgery	After surgery				
		1st week	1st month	3rd month	6th month	1st year
A	25.34 ± 1.24	21.40 ± *1*.50*	21.90 ± *1*.30*	21.93 ± *1*.12*	22.30 ± *1*.24*	23.34 ± *1*.04*
B	22.49 ± *0*.*69*	22.30 ± *0*.67*	22.35 ± *0*.66*	22.38 ± *0*.68*	22.40 ± *0*.69*	23.40 ± *0*.73*
C	32.19 ± *1*.*09*	28.68 ± *1*.16*	28.90 ± *1*.28*	29.03 ± *1*.35*	28.65 ± *1*.35*	29.15 ± *1*.45*
D	30.37 ± *1*.*66*	28.45 ± *3*.*06*	27.40 ± *0*.99*	27.40 ± *0*.93*	27.70 ± *1*.05*	27.75 ± *1*.43*
E	36.88 ± *1*.*45*	31.93 ± *1*.21*	31.90 ± *1*.24*	32.35 ± *1*.43*	32.45 ± *1*.62*	33.15 ± *1*.17*
F	39.88 ± *3*.*16*	37.85 ± *2*.96*	38.03 ± *3*.05*	38.15 ± *2*.72*	37.85 ± *2*.35*	38.10 ± *2*.65*

Abbreviations: (A) The part of the hand between the thumb and the index finger; (B) wrist; (C) the median of the forearm; (D) elbow through olecranon; (E) the median of the upper arm; (F) the root of the upper arm. *p < 0.05, based on a paired t-test.

**Table 2:** The circumference of the affected upper limb after physiotherapy ($\overline{{\rm{x}}}$ ± s, cm).

The 6 points	Basement circumference	After surgery				
		1st week	1st month	3rd month	6th month	1st year
A	22.49 ± *0*.*69*	22.30 ± *0*.*67*	22.35 ± *0*.*66*	22.38 ± *0*.*68*	22.40 ± *0*.*69*	23.40 ± *0*.73*
B	18.25 ± *0*.*88*	18.10 ± *0*.*65*	18.33 ± *0*.*70*	18.35 ± *0*.*88*	18.26 ± *1*.*13*	19.00 ± *1*.*45*
C	28.03 ± *1*.*59*	28.07 ± *1*.*61*	27.87 ± *1*.*69*	27.87 ± *1*.*57*	28.05 ± *2*.*06*	29.00 ± *2*.*22*
D	26.65 ± *1*.*22*	27.10 ± *0*.*93*	27.83 ± *0*.*80*	28.18 ± *0*.*81*	28.35 ± *0*.85*	28.70 ± *0*.97*
E	31.04 ± *1*.*56*	30.87 ± *1*.*42*	31.60 ± *1*.*88*	32.35 ± *2*.*32*	32.80 ± *2*.*65*	33.55 ± *3*.05*
F	35.29 ± *2*.*54*	36.10 ± *2*.*07*	36.70 ± *1*.96*	37.00 ± *1*.56*	37.60 ± *1*.*82*	37.90 ± *1*.69*

Abbreviations: (A) The part of the hand between the thumb and the index finger; (B) wrist; (C) the median of the forearm; (D) elbow through olecranon; (E) the median of the upper arm; (F) the root of the upper arm. *p < 0.05, based on a paired t-test.

Results of the patient questionnaire on their subjective assessment about their affected limbs are shown in Table 3. Patients who underwent surgery felt their oedema had improved to a significantly greater degree than did patients in the physiotherapy group in terms of swelling (*p* = 0.008), pain and numbness (*p* = 0.03), and limitations in range of motion (*p* = 0.021). In our survey, one patient in the surgery group and two in the physiotherapy group reported that after modified radical mastectomy, the affected limb had a delayed healing time, even for minor injuries such as scratches or bruises. However, neither reconstructive surgery nor physiotherapy mitigated this symptom. Based on the 6 points circumferences and the answers to the questionnaire, we found that the swelling increased by less than 0.3–0.5 cm in the second year and stabilised in the third year for both the surgery and physiotherapy groups. None of the 10 patients in the TRAM/DIEP group experienced oedema or pain in the lower extremity in the donor area after lymphatic TRAM/DIEP surgery. Delayed abdominal wound healing was observed after 8 weeks in two patients in the surgery group, but neither required further surgeries for this reason.

**Table 3:** Questionnaire of patients’ affected limb sensations and motion range changes after therapy.

Patients’ subjective assessment	Therapy efficiency	Patient quantity		*p* ^a^ value
		Surgery Group n (%)	Physiotherapy Group n (%)	
Swelling				0.008
	excellent	3 (30)	0 (0)	
	Effective	6 (60)	2 (20)	
	invalid	1 (10)	6 (60)	
	aggravate	0 (0)	2 (20)	
Pain and numbness				0.03
	excellent	5 (50)	0 (0)	
	effective	4 (40)	4 (40)	
	invalid	1 (10)	5 (50)	
	aggravate	0 (0)	1 (1)	
Motion range				0.021
	excellent	2 (20)	0 (0)	
	effective	8 (80)	4 (40)	
	invalid	0 (0)	4 (40)	
	aggravate	0 (0)	2 (2)	

^a^p-value is based on Fisher’s exact test.

## Discussion

Lymphoedema is the most prominent nonfatal complication associated with breast cancer. Treatment-related factors are the primary cause of BRCL, especially surgical treatments involved with axillary lymph node dissection and radiotherapy^5–10^. Patients with lymphoedema report some of the following symptoms: tingling (35%), numbness (62–67%), pain (30–68%), weakness (67%), stiffness (60%), decreased range of motion (40–45%)^3, 6^, and Stewart syndrome, a rare and deadly disease^11, 12^.

Presently, there is no standard treatment for lymphoedema. Many types of treatment have been employed, including surgical and non-surgical strategies. Non-surgical treatments include compression with a pneumatic pump, elastic sleeves or gloves, support garments or wrapping, massage therapy, arm elevation, antibiotics, drug therapy, physical therapy, or avoidance of certain activities^13^. The majority (96%) of women who received treatment reported receiving multiple treatments^14^. Another exploratory therapeutic strategy for BCRL is surgery using microsurgical techniques to transfer vascularised lymph nodes individually^4, 15, 16^, with the goal of reconnecting lymph drainage. The donor sites for vascularised lymph node transfer include groin, submental, supraclavicular, and the jejunal mesenteric areas^4, 16–18^. The inguinal nodes are the most commonly used donor sites^19^.

We chose TRAM/DIEP flap reconstruction combined with the inguinal lymph node donation. Breast reconstruction and lymphoedema were thus addressed at the same time. In our study, we found that patients who accepted reconstructive surgery after ALND and radiotherapy had better outcomes than did patients receiving only physical therapy for upper-limb lymphoedema. The mechanism for alleviating lymphoedema in our surgical strategy was based on two effects. First, the transfer of lymph nodes could induce endogenous lymphatic growth factor expression, leading to vessel regeneration^20–22^. The lymphatic vessel growth factor, Vascular endothelial growth factor-C, plays an important role in this process^20, 23^. Second, widely removing all old scar tissue from the axilla is also likely to release axillary veins, enabling the growth of new vessels into this area. Additionally, our transferred lymph tissues were encircled the superficial circumflex vessel pedicle, which may have a blood supply sufficient for lymph tissue survival, and these lymph tissues were connected to a lower abdominal flap to facilitate good lymph reflux. Other researchers investigating lymphatic TRAM/DIEP have also found that the lymphatic flap is very useful for relieving lymphoedema^22^. However, like us, they were unable to use this surgery to cure the lymphoedema completely, although the lymphatic TRAM/DIEP flap minimised lymphoedema to some degree. Because surgical and non-surgical strategies for lymphoedema have not yet been perfected, prevention of lymphoedema is more important than its treatment and early diagnosis and treatment of lymphoedema is believed to lead to better outcomes^14^.

In our study, we directly compared the efficacy of the lymphatic TRAM/DIEP flap to physiotherapy and found that lymphatic TRAM/DIEP is more effective than physiotherapy for the treatment of post-mastectomy lymphedema, and offers the additional benefit of simultaneous breast reconstruction.

## Methods

### Patient selection criteria

Approval for the study was granted by the ethics committee of the Third Affiliated Hospital of Kunming Medical University. We confirmed that all methods were performed in accordance with the relevant guidelines and regulations. All of the patients sighed informed consent. After completion of breast surgery and radiotherapy by both surgeons and radiation oncologists at this hospital, patients were assessed at regular intervals, ranging from 3 months to 1 year. Circumferential limb measurements were taken at six anatomical points: the palm of the hand between the thumb and the index finger, the wrist, the median of the forearm, the elbow through the olecranon, and the median and the root of the upper arm. According to the swelling severity, three levels of BCRL were classified. Mild oedema was diagnosed when the circumference of the affected limb was 0–3 cm larger than that of the intact side or when the swelling was limited to the proximal side of the upper limb; moderate oedema was diagnosed when the circumference of the affected limb was 3.1–6 cm larger than that of the intact side or the swelling extended to the forearm and palm; and severe oedema was diagnosed when the circumference of the affected limb was ≥6 cm larger than that of the intact side or the oedema caused symptomatic restriction of arm movement. Persistent oedema was only scored if the symptoms were not resolved at subsequent visits. For this study, we only enrolled patients with persistent moderate to severe oedema related to BCRL, which is oedema that lasts more than half a year. Twenty patients with BCRL after modified radical mastectomy were enrolled between November 2012 and October 2014: 12 were enrolled between November 2012 and November 2013, and 8 were enrolled between December 2013 and October 2014. BCRL patients willing to undergo breast reconstruction were enrolled in the lymphatic TRAM/DIEP group (n = 10), and BCRL patients unwilling to undergo breast reconstruction were enrolled in physiotherapy (n = 10).

None of these 20 patients had previously undergone surgery with a TRAM flap, abdominal suction-assisted lipectomy, or abdominal surgery leading the deep inferior epigastric vessels divided or damaged. They also had none significant medical co-morbidities that make them poor surgical candidates. In the surgery group, 1 patient was a cigarette smoker and 2 were obese. These 3 received a TRAM flap, whereas the other 7 were treated using the DIEP strategy. Physiotherapy for patients in the physical therapy group included pneumatic compression and decongestive bandage depression.

### Reconstructive surgery procedure

#### Preparation

Ultrasound was used to detect the transverse rectus abdominis myocutaneous/deep inferior epigastric perforator vessels, internal mammary vessels, and subscapular vessels to evaluate the vessels for anastomosis. Both limbs were evaluated with circumference measured at six positions.

#### Operation 1: Chest, axillary, and abdominal area procedure

**Figure 2:**
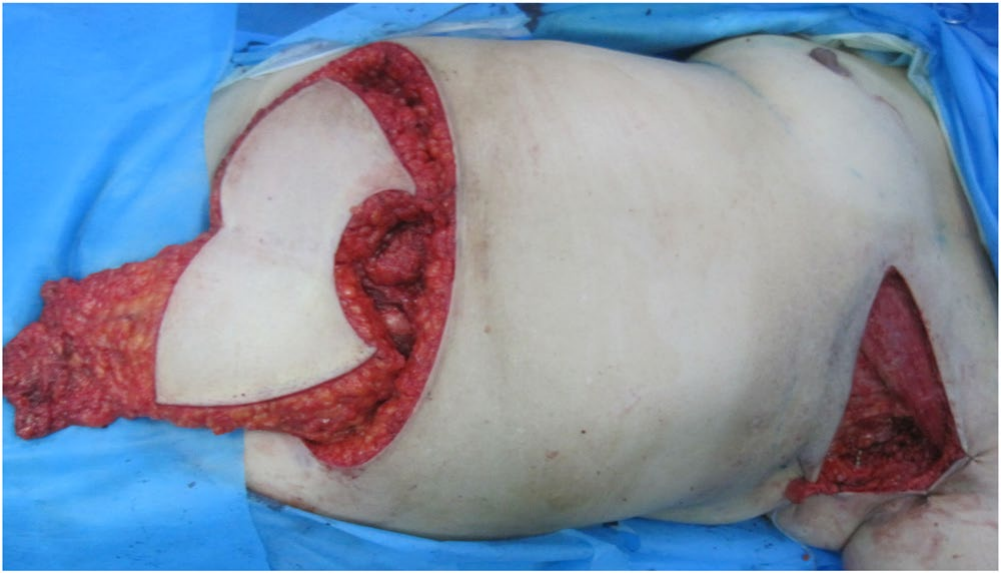
The lymphatic TRAM/DIEP flap. The flap for simultaneous breast reconstruction and lymph node transferring included lymphatic tissue surrounding the superficial circumflex iliac vessels.

Scar tissue on the chest, including the scar surrounding axillary vessels and subscapular vessels, was ablated until normal tissue was reached. The circumflex scapular vessels and thoraco-dorsal vessels were evaluated as recipient vessels. The released scar tissue was sent for pathologic evaluation. TRAM or DIEP surgery was performed according to a standard procedure^24^. The key procedure for lymphatic TRAM or DIEP was the harvesting of donor lymph tissue and the vessels supplying it. We harvested lymph tissue from the inguinal region surrounding the superficial circumflex iliac vessels. The lymphatic tissue contained lymph nodes, lymphatic vessels, and fat from the groin area surrounding the superficial circumflex iliac vessels (superficial circumflex iliac artery) or its perforators (Fig. 2).

#### Operation 2: Vessel anastomosis and donor lymph tissue insetting

If the flap supplying the vessels was ipsilateral to the donor lymph tissue, it was only necessary to anastomose the flap vessels to the recipient vessels. However, if the flap supplying the vessels and donor lymph tissue was on a different side, both the flap supplying the vessels and the lymph tissue supplying vessels needed to be anastomosed to the recipient vessels. This situation occurred for 1 patient with lymphatic DIEP. For this patient, when we first harvested lymph tissue from the inguinal region on the left side, we found that the ipsilateral deep inferior epigastric perforator vessels were not sufficient to supply the flap, so we decided to use the contralateral deep inferior epigastric perforator vessels to supply the flap. In this case, we anastomosed the right side deep inferior epigastric perforator vessels to the subscapular vessels and the left side superficial circumflex iliac vessels in the donor lymph tissue to the branch of the superficial circumflex iliac vessels. After the recipient and donor vessels were anastomosed, the lymph tissue from the iliac region was covering the surface of the axillary vessels.

#### Postoperative treatment and follow-up strategy

For the surgery group, patients were placed in knee and hip flexion for 2 weeks to minimise tension at the abdominal site. The flap was monitored every 2 hours by nursing or surgical staff for the first 3 days. Decongestive bandage compression, avoiding the area with transplanted axillary lymph nodes and anastomosed vessels, was used continuously for the first year. In the physiotherapy group, in addition to the decongestive bandage compression, which was used as described for the surgery group, pneumatic compression was used for 20 min twice a day for the first 6 months and for 20 min every 2 days for the second 6 months. During the follow-up period, the upper limbs were measured at 6 points circumferences of the affected limb and patient responses to a questionnaire were used to evaluate improvement in lymphoedema at 7 days, 1 month, 3 months, 6 months, and 1 year following surgery. The questionnaire was patients’ subjective assessment about the affected limb, including swelling feeling, pain and numbness, and motion range. The criteria for evaluating treatment efficacy levels were as follows: significant efficacy: the circumference of the affected limb overall had totally recovered to that of the contralateral limb; moderate efficacy: more than or equal to 4 points of the whole affected limb circumference improved by one level; slight efficacy: the circumference of the affected limb in its entirety decreased but not by an entire level or no more than 4 points improved by one level; and no efficacy: the circumference of the affected limb did not change or it worsened. The measures of limb circumference and range of motion were carried out once by the same physiotherapist.

### Statistics

Propensity score matching was used to match patients between groups to balance their baseline data. Data were analysed by SPSS 21. Student’s t-test was used for measurement data, and Fisher’s exact test and logistic regression analyses were used for count data. At this manuscript, the follow up time was end by August 2016. The 12 patients enrolled during November 2012 to November 2013 had complete second year follow up data but not third year follow up data. And the 8 patients enrolled during December 2013 to October 2014 did not have complete second year follow up data. So, in this manuscript, only the first year follow up data was analyzed by statistic method. The second and the third year data were just described briefly.

## References

[1] Torre LA (2015). Global cancer statistics, 2012. CA Cancer J Clin.

[2] DiSipio T, Rye S, Newman B, Hayes S (2013). Incidence of unilateral arm lymphoedema after breast cancer: a systematic review and meta-analysis. Lancet Oncol.

[3] Hayes SC, Janda M, Cornish B, Battistutta D, Newman B (2008). Lymphedema after breast cancer: incidence, risk factors, and effect on upper body function. J Clin Oncol.

[4] Kibar, S., Dalyan Aras, M. & Unsal Delialioglu, S. The risk factors and prevalence of upper extremity impairments and an analysis of effects of lymphoedema and other impairments on the quality of life of breast cancer patients. Eur J Cancer Care (Engl) (2016). 10.1111/ecc.12433 26764197

[5] Coen JJ, Taghian AG, Kachnic LA, Assaad SI, Powell SN (2003). Risk of lymphedema after regional nodal irradiation with breast conservation therapy. Int J Radiat Oncol Biol Phys.

[6] Das N (2015). Treatment-related risk factors for arm lymphedema among long-term breast cancer survivors. J Cancer Surviv.

[7] Williams AF, Franks PJ, Moffatt CJ (2005). Lymphoedema: estimating the size of the problem. Palliat Med.

[8] Seifart U (2007). Lymphedema in patients with breast cancer–a consensus regarding diagnostics and therapy in patients with postoperative lymphedema after primary breast cancer. Rehabilitation (Stuttg).

[9] Vignes S, Arrault M, Dupuy A (2007). Factors associated with increased breast cancer-related lymphedema volume. Acta Oncol.

[10] Meeske KA (2009). Risk factors for arm lymphedema following breast cancer diagnosis in Black women and White women. Breast Cancer Res Treat.

[11] Young RJ, Brown NJ, Reed MW, Hughes D, Woll PJ (2010). Angiosarcoma. Lancet Oncol.

[12] Sharma A, Schwartz RA (2012). Stewart-Treves syndrome: pathogenesis and management. J Am Acad Dermatol.

[13] Yamamoto T (2008). Study of edema reduction patterns during the treatment phase of complex decongestive physiotherapy for extremity lymphedema. Lymphology.

[14] Logan V, Barclay S, Caan W, McCabe J, Reid M (1996). Knowledge of lymphoedema among primary health care teams: a questionnaire survey. Br J Gen Pract.

[15] Becker C, Assouad J, Riquet M, Hidden G (2006). Postmastectomy lymphedema: long-term results following microsurgical lymph node transplantation. Ann Surg.

[16] Cheng MH (2012). A novel approach to the treatment of lower extremity lymphedema by transferring a vascularized submental lymph node flap to the ankle. Gynecol Oncol.

[17] Coriddi, M., Skoracki, R. & Eiferman, D. Vascularized jejunal mesenteric lymph node transfer for treatment of extremity lymphedema. Microsurgery (2016). 10.1002/micr.30037 26892278

[18] Althubaiti GA, Crosby MA, Chang DW (2013). Vascularized supraclavicular lymph node transfer for lower extremity lymphedema treatment. Plast Reconstr Surg.

[19] Scaglioni, M. F. *et al*. Comprehensive review of vascularized lymph node transfers for lymphedema: Outcomes and complications. Microsurgery (2016). 10.1002/micr.30079 27270748

[20] Saaristo A (2006). Vascular endothelial growth factor-C accelerates diabetic wound healing. Am J Pathol.

[21] Tammela T, Alitalo K (2010). Lymphangiogenesis: Molecular mechanisms and future promise. Cell.

[22] Saaristo AM (2012). Microvascular breast reconstruction and lymph node transfer for postmastectomy lymphedema patients. Ann Surg.

[23] Paavonen K, Puolakkainen P, Jussila L, Jahkola T, Alitalo K (2000). Vascular endothelial growth factor receptor-3 in lymphangiogenesis in wound healing. Am J Pathol.

[24] Chang DW (2012). Breast Reconstruction with Microvascular MS-TRAM and DIEP Flaps. Arch Plast Surg.

